# Genome‐wide association study for reproductive traits in a Large White pig population

**DOI:** 10.1111/age.12638

**Published:** 2018-02-07

**Authors:** Y. Wang, X. Ding, Z. Tan, K. Xing, T. Yang, Y. Pan, Y. Wang, S. Mi, D. Sun, C. Wang

**Affiliations:** ^1^ Key Laboratory of Animal Genetics and Breeding of Ministry of Agriculture National Engineering Laboratory of Animal Breeding College of Animal Science and Technology China Agricultural University Beijing 100094 China; ^2^ Beijing Shunxin Agriculture Co., Ltd. Beijing 101300 China; ^3^ Beijing Liuma Pig Breeding Technology Co., Ltd. Beijing 101308 China

**Keywords:** candidates, genetic mechanism, GWAS, single nucleotide polymorphism, phenotypic variance

## Abstract

Using the PorcineSNP80 BeadChip, we performed a genome‐wide association study for seven reproductive traits, including total number born, number born alive, litter birth weight, average birth weight, gestation length, age at first service and age at first farrowing, in a population of 1207 Large White pigs. In total, we detected 12 genome‐wide significant and 41 suggestive significant SNPs associated with six reproductive traits. The proportion of phenotypic variance explained by all significant SNPs for each trait ranged from 4.46% (number born alive) to 11.49% (gestation length). Among them, 29 significant SNPs were located within known QTL regions for swine reproductive traits, such as corpus luteum number, stillborn number and litter size, of which one QTL region associated with litter size contained the *ALGA0098819* SNP for total number born. Subsequently, we found that 376 functional genes contained or were near these significant SNPs. Of these, 14 genes—*BHLHA15*,* OCM2*,* IL1B2*,* GCK*,* SMAD2*,* HABP2*,* PAQR5*,* GRB10*,* PRELID2*,* DMKN*,* GPI*,* GPIHBP1*,* ADCY2* and *ACVR2B—*were considered important candidates for swine reproductive traits based on their critical roles in embryonic development, energy metabolism and growth development. Our findings contribute to the understanding of the genetic mechanisms for reproductive traits and could have a positive effect on pig breeding programs.

In pig production systems, reproductive traits, such as total number born (TNB), number born alive (NBA), litter birth weight (LBW), average birth weight (ABW), gestation length (GL), age at first service (AFS) and age at first farrowing (AFF), play key roles in production efficiency and economic profits. Traditional breeding technologies based on best linear unbiased prediction (Holm *et al*. 2004) is limited for significant genetic improvement of these reproductive traits due to their low heritability (Chen *et al*. 2003). In addition, piglet records can be collected for sows only later in life. Thus, for improved breeding programs, it is essential to better understand the genetic determinants of these traits. Candidate genes and quantitative trait loci (QTL) for reproductive traits were identified in previous studies (Onteru *et al*. 2009). Until now, a total of 405 QTL had been found on different swine chromosomes for TNB, NBA, LBW, ABW and GL (http://www.animalgenome.org/cgi-bin/QTLdb/SS/index, Apr 27, 2017).

Compared with previous methods, genome‐wide association studies (GWASs) provide a more powerful strategy for genetic dissection and have been performed with domestic animals for various economic traits (Zhang *et al*. 2013). To date, several GWASs for pig reproductive traits, such as number of teats (Verardo *et al*. 2016), litter size (Sell‐Kubiak *et al*. 2015) and ovulation rate (He *et al*. 2017), have been conducted, but knowledge about the complex genetic mechanism for reproductive performance still remains insufficient. Therefore, the aim of this study was to detect novel genetic variants and identify candidate genes for reproductive traits by performing a GWAS in a Large White pig population.

A total of 1207 Large White pigs from the Beijing Shunxin Agriculture Co., Ltd. and Beijing Liuma Pig Breeding Technology Co., Ltd. pig breeding farms (Beijing, China) were genotyped using the GeneSeek PorcineSNP80 BeadChip. Using plink software (Purcell *et al*. 2007), DNA samples with genotyping call rates less than 90% were removed, and we also excluded SNPs with call rates less than 90%, minor allele frequencies less than 0.03 or Hardy–Weinberg equilibrium *P*‐value < 1.00E‐06 in SNPs with no position information or located on sex chromosomes were also excluded from the dataset. Missing genotypes were imputed using beagle v4.0 (Browning & Browning 2009) based on information from remaining SNP genotypes using, and SNPs with *R* ^2^ > 0.3 (Browning & Browning 2007) were retained. After quality control, 1198 individuals and 51 443 SNPs qualified for the study.

The phenotypic data for first parity were collected from 2010 to 2015, and statistics for the phenotypes of seven reproductive traits were calculated (Table S1). TNB, NBA, LBW, ABW and GL were approximately normally distributed, but for AFS and AFF, phenotype values were converted using the rntransform function in the genabel r package (Aulchenko *et al*. 2007).

To better control population structure, we first conducted a principal components analysis to reduce spurious associations derived from the presence of individual relatedness. All autosomal SNPs were pruned using the indep‐pairwise option in plink software, using a window size of 25 SNPs, a step of five SNPs and an *r* ^2^ threshold of 0.2 (Gu *et al*. 2011), which resulted in 6993 independent SNPs.

The genome‐wide association study was implemented with a mixed model approach using gemma software (Zhou & Stephens 2012) for each trait in the first parity. The centered relatedness matrix was calculated using all autosomal markers, and a Wald test was performed for each SNP against the null hypothesis of *g *=* *0. The statistical model used is as follows:

y=Wα+xβ+u+ε

where **y** is an *n*×1 vector of phenotype values for all individuals; **W** is an *n×c* matrix of covariates (fixed effects that contain the first PC, herd, farrowing season and a column of 1s); **α** is a *c*×1 vector of the corresponding coefficients including the intercept; **x** is an *n×*1 vector of genotypes of a marker at the locus tested; *β* is the effect size of the marker; **u** is an *n×*1 vector of random polygenic effects with a covariance structure as **ɛ**∼*N*(0, **K** *V*_*g*_), where **K** is an *n×n* genetic relatedness matrix and *V*_*g*_ is the polygenic additive variance; and **ɛ** is an *n×*1 vector of residual errors with **ɛ**∼*N*(0, **I** *V*_*e*_), where **I** is *n* **×** *n* identity matrix and *V*_*e*_ is the residual variance component.

To properly decide the thresholds for genome‐wide significant/suggestive associations, we calculated the effectively independent tests based on the estimated number of independent markers and linkage disequilibrium blocks for autosome markers (Nicodemus *et al*. 2005). A linkage disequilibrium block was defined as a set of adjacent SNPs with pairwise *r* ^2^ values greater than 0.40 (Gu *et al*. 2011). A total of 11 315 effectively independent tests was suggested, following Lander & Kruglyak (1995), and the threshold *P*‐value for genome‐wide significance association was 4.42E–6 (0.05/11 315) and for suggestive association was 8.84E–5 (1/11 315). The genomic inflation factor λ was calculated using genabel packages. In addition, gcta software (Yang *et al*. 2011) was used to calculate phenotypic variances explained by significant SNPs.

The functional genes containing or near the identified significant SNPs, less than 1 Mb away from significant SNPs, were selected based on the *Sus scrofa* 10.2 genome assembly. Further functional annotation was carried out based on the NCBI database (https://www.ncbi.nlm.nih.gov/), and Gene Ontology analysis was conducted using DAVID Bioinformatics Resources (http://david.abcc.ncifcrf.gov/).

As a result, 53 SNPs, including 12 genome‐wide significant (Table 1) and 41 suggestive significant (Table S2) SNPs, were detected for TNB, NBA, LBW, GL, AFS and AFF on SSC1, 2, 3, 4, 5, 6, 9, 10, 11, 13, 14, 15, 16 and 18 (there were no significant SNPs detected for LBW). The lambda values were 1.01, 1.02, 1.02, 1.01, 1.01 and 1.01 for each trait respectively, which meant lower population stratification (Price *et al*. 2010). Manhattan plot and quantile–quantile plots for TNB, NBA and ABW are shown in Fig. 1, and the plots for GL, AFS and AFF are shown in Fig. S1.

**Table 1:** Genome‐wide significant SNPs for five reproductive traits

Trait	SNP	Chr	Position (bp)	*P*‐value	MAF	*β*	CPV% (SE)	Nearest gene/candidate gene^a^	Location (bp)^b^
TNB	*WU_10.2_2_162527469*	2	162 527 469	9.72E–07	0.27 (C/A)	−0.59	2.18 (0.03)	*IFITM2*	174 050
TNB	*WU_10.2_3_44631648*	3	44 631 648	1.19E–06	0.31 (G/A)	−0.55	2.09 (0.03)	*BARX1*/***IL1B2***	23 864/**53 8271**
TNB	*WU_10.2_3_44862084*	3	44 862 084	2.83E–06	0.27 (G/A)	−0.57	2.01 (0.03)	*BARX1*/***IL1B2***	254 300/**307 835**
TNB	*ALGA0098819*	18	56 535 534	3.46E–06	0.17 (A/G)	−0.62	1.72 (0.03)	*LOC102165380*/***GCK***	within/**617 340**
NBA	*WU_10.2_3_44631648*	3	44 631 648	9.07E–07	0.31 (G/A)	−0.53	2.08 (0.03)	*BARX1/**IL1B2***	23 864/**538 271**
NBA	*ALGA0098819*	18	56 535 534	3.10E–06	0.17 (A/G)	−0.61	1.79 (0.03)	*LOC102165380/**GCK***	within/**617 340**
NBA	*WU_10.2_2_162527469*	2	162 527 469	4.42E–06	0.27 (C/A)	−0.53	1.93 (0.03)	*IFITM2*	174 050
GL	*ALGA0061535*	11	25 305 148	1.42E–06	0.05 (A/G)	−1.04	2.26 (0.03)	*AKAP11*	99 469
GL	*WU_10.2_4_1247716*	4	1 247 716	3.32E–06	0.16 (A/G)	−0.66	1.93 (0.03)	*ZC3H3/**GPIHBP1***	14 516/**96 877**
GL	*ASGA0023643*	4	1 166 037	3.87E–06	0.16 (A/G)	−0.66	1.88 (0.03)	*MAFA*	7303
AFS	*ALGA0111336*	16	80 445 920	2.23E–06	0.38 (A/C)	−0.22	2.26 (0.03)	***ADCY2***	**within**
AFF	*ALGA0111336*	16	80 445 920	3.53E–06	0.38 (A/C)	−0.22	2.19 (0.03)	***ADCY2***	**within**

Chr, swine Chromosome; MAF, allele frequency of first listed marker; *β*, allele substitution effect; CPV% (SE), contribution to phenotypic variance (standard error); TNB, total number born; NBA, number born alive; GL, gestation length; AFS, age at first service; AFF, age at first farrowing.

a Gene names in bold type represent candidate genes with less than 1.0 Mb of the SNPs.

b Locations in bold type represent the distance between a significant SNP and the candidate gene.

**Figure 1:**
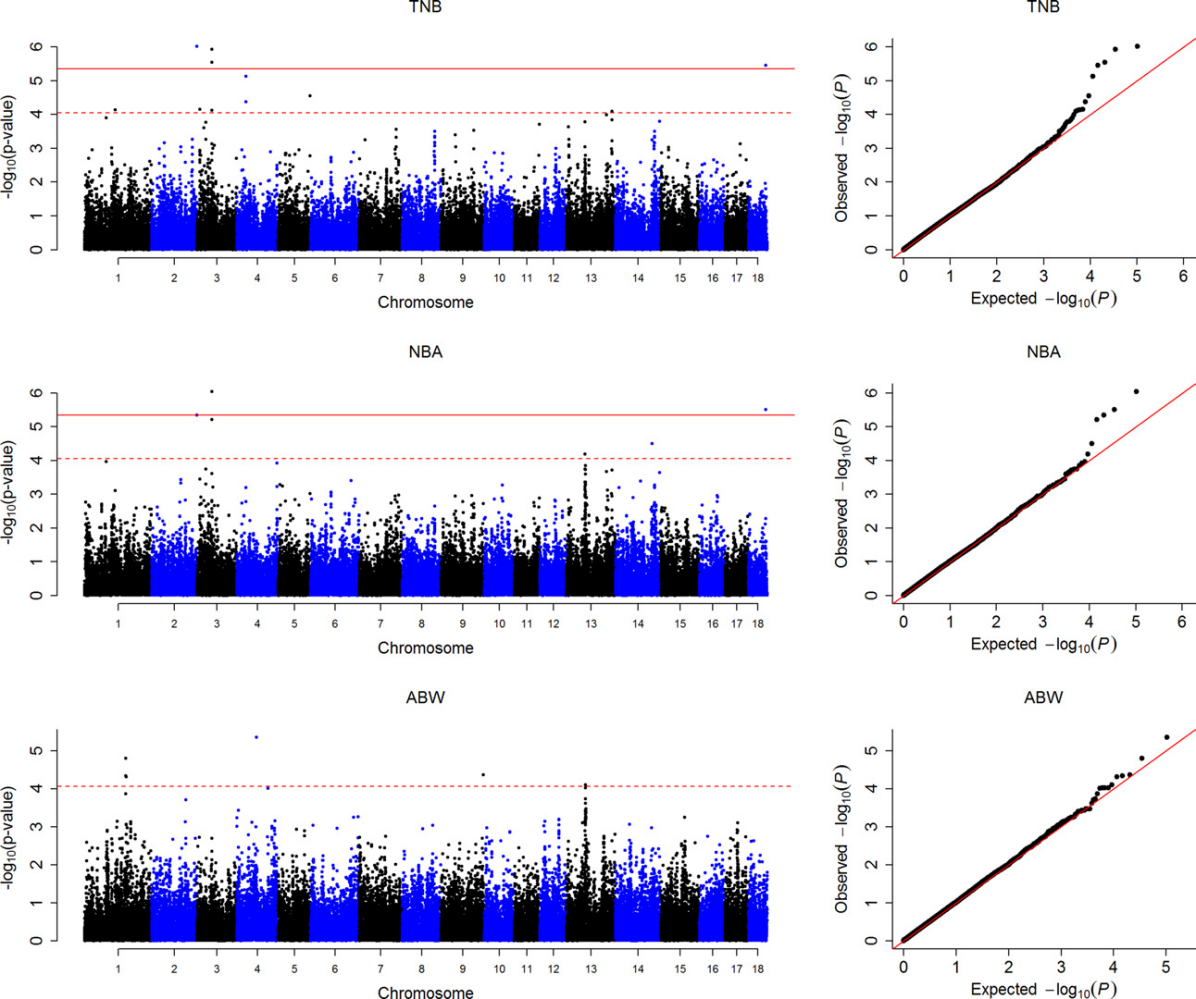
Manhattan plots and quantile–quantile (Q–Q) plots of the observed *P*‐values for total number born (TNB), number born alive (NBA) and average birth weight (ABW**)**. The horizontal red and red dashed lines in the Manhattan plots indicate the genome‐wide (4.42 × 10^−6^) and suggestive significance (8.84 × 10^−5^) thresholds respectively. The Q–Q plots show the observed –log_10_‐transformed *P*‐values (*y*‐axis) and the expected –log_10_‐transformed *P*‐values (*x*‐axis).

The phenotypic variance explained by each significant SNP ranged from 1.24% to 2.26% (Tables 1 & S2). The phenotypic variance explained by all significant SNPs was 6.77% (SE = 0.03) and 4.46% (SE = 0.03) for TNB and NBA respectively. Of note, among them, individuals with genotype AA at SNP *WU_10.2_2_162527469* on SSC2 had higher TNB than did those with genotypes AC or CC, whereas individuals with genotype GG at SNP *WU_10.2_3_44631648* on SSC3 showed lower NBA than did those with genotypes AG and AA (Table S3). For AFS and AFF, the phenotypic variance explained by all significant SNPs was 7.98% (SE = 0.048) and 9.38% (SE = 0.05) respectively. The peaking SNP *ALGA0111336* on SSC16 revealed that individuals with genotype CC had higher AFS and AFF than did those with other genotypes (Table S3). In addition, for two other traits, ABW and GL, the phenotypic variance explained by all significant SNPs was equal to 7.11% (SE = 0.05) and 11.49% (SE = 0.05) respectively.

A total of 376 different functional genes (Table S4) that contained or were near the significant SNPs were selected. Combined with Gene Ontology analysis (Table S5), 14 genes with biological functions, such as carbohydrate metabolism, lipid metabolism and embryonic development, were selected as promising candidates for swine reproductive traits (Tables 1 and S2).

For TNB and NBA, we selected six functional genes. Both the *IL1B2* and *GCK* genes simultaneously associated with two traits. The *IL1B2* gene promotes follicular growth, corpus luteum formation and embryo development (Ross *et al*. 2003). The *GCK* gene encodes an enzyme that regulates glucose level (Muller *et al*. 2014) and affects the supplement of fetal energy substrate. Three other genes—*BHLHA15*,* OCM2* and *SMAD2*—associated with TNB. The *BHLHA15* gene has a critical role in mouse embryonic development, especially in gastrulae and plantule (Pin *et al*. 2000). The *OCM2* gene contributes to the transport of calcium and affects fetal skeletal mineralization (Belkacemi *et al*. 2002). We also selected the *SMAD2* gene, which promotes actions of follistatin on blastocyst development in early embryogenesis (Zhang *et al*. 2015). Furthermore, the *HABP2* gene is another candidate gene for NBA and plays a role in the integrity of mouse cumulus extracellular matrix (Chen *et al*. 1993).

The *PAQR5* and *GRB10* genes were selected as important candidates for ABW. The *PAQR5* gene promotes fast regulation of progesterone through a combination of specific receptors on the cell membrane (Thomas 2008), and the expression of *GRB10* in the placenta directly regulates placental size and efficiency in mouse (Charalambous *et al*. 2010).

Four genes—namely *PRELID2*,* DMNK*,* GPI* and *GPIHBP1*—associated with GL. *PRELID2* is involved in mouse embryogenesis during mid‐later gestation (Gao *et al*. 2009), whereas *DMNK* plays a role in the process of embryonic implantation (Paria *et al*. 2002). The function of the *GPI* gene is similar to that of *GCK:* regulating glucose homeostasis. Like glucose, lipids play critical roles in fetal growth, and the *GPIHBP1* gene participates in the transportation of lipids, including cholesterol, triglycerides and other lipids (Herrera 2002).

A common candidate gene associated with AFS and AFF is the *ADCY2* gene, which contained the peak SNP *ALGA0111336* and is involved in catalyzing the synthesis of the secondary messenger cyclic adenosine monophosphate (cAMP), which promotes the functions of follicle stimulating hormone and luteinizing hormone on ovaries (Richards *et al*. 1995). Another candidate for AFF, *ACVR2B*, plays an important role in the production of estrogen and progesterone, oocyte maturation and follicle stimulating hormone receptor expression (Findlay 1993).

The sample size in this study, consisting of 1207 individuals, was larger than that used by Schneider *et al*. (2012), who used a composite population with 1152 individuals, and Onteru *et al*. (2011, 2012), who used 683 and 818 commercial sows respectively. Our study enriches the understanding of genetic mechanisms for reproductive traits, especially for AFS and AFF in pigs.

Compared with previous studies, there were 29 significant SNPs located on known QTL regions for reproductive traits (Table S6), such as number of teats (Verardo *et al*. 2016), number of stillborn and litter size (Onteru *et al*. 2012). One QTL region associated with litter size (SSC18, 52.3–77.6 MB) contained the *ALGA0098819* SNP for TNB; the *GCK* gene is located in this QTL region.

In summary, 53 significant SNPs were detected to be associated with six reproductive traits. Further, 14 functional genes were identified to be important candidates for TNB, NBA, ABW, GL, AFS and AFF, namely, *BHLHA15*,* OCM2*,* IL1B2*,* GCK*,* SMAD2*,* HABP2*,* PAQR5*,* GRB10*,* PRELID2*,* DMKN*,* GPI*,* GPIHBP1*,* ADCY2* and *ACVR2B*. Our findings provide important knowledge on the understanding of genetic architecture for swine reproductive traits.

## Supporting information


**Figure S1** Manhattan plots and quantile–quantile plots of the observed *P*‐values for gestation length (GL), age at first service (AFS) and age at first farrowing (AFF).

**Table S1** Descriptive statistics of reproductive traits in the Large White population.

**Table S2** Suggestive significant SNPs for six reproductive traits.

**Table S3** Genotype–phenotype correlations of the most significant SNPs for four reproductive traits

**Table S4** Annotated genes with less than 1 Mb of significant SNPs.

**Table S5** Significant Gene Ontology terms for five reproductive traits.

**Table S6** Significant SNPs located in known QTL regions for reproductive traits.
