# Supplementary material for: Genome‐wide association study for reproductive traits in a Large White pig population
Source: Anim Genet. 2018 Feb 7;49(2):127–31. doi: 10.1111/age.12638 (PMC5873431; doi:10.1111/age.12638)

Figure S1 Manhattan plots and quantile–quantile plots of the observed *P*-values for gestation length (GL), age at first service (AFS) and age at first farrowing (AFF).

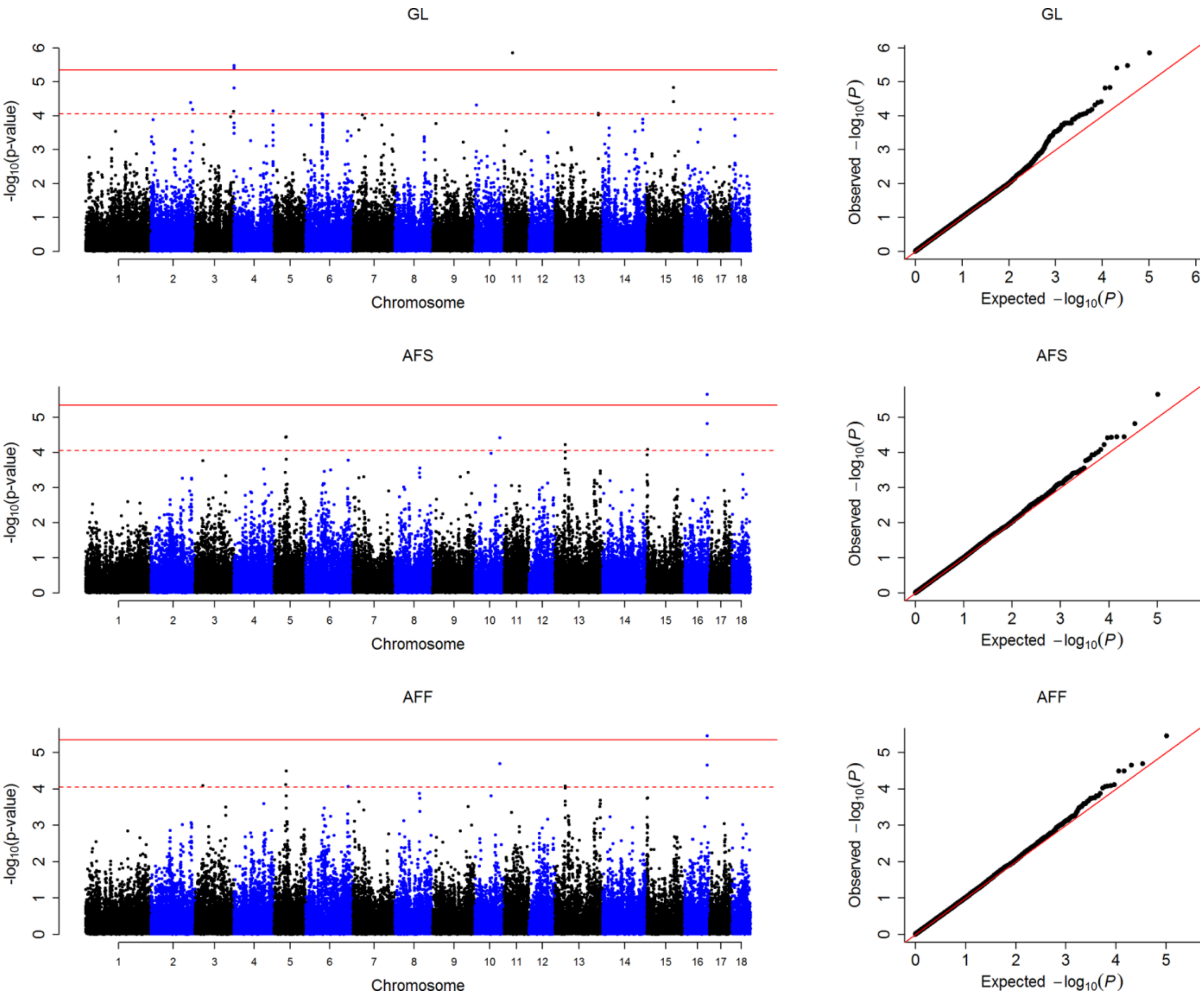

Supplement: Supplementary file 1 — Figure S1 Manhattan plots and quantile–quantile plots of the observed P‐values for gestation length (GL), age at first service (AFS) and age at first farrowing (AFF). [file AGE-49-127-s001.pdf]
