# Supplementary material for: Genome‐wide association study for reproductive traits in a Large White pig population
Source: Anim Genet. 2018 Feb 7;49(2):127–31. doi: 10.1111/age.12638 (PMC5873431; doi:10.1111/age.12638)
Supplement: Supplementary file 2 — Table S1 Descriptive statistics of reproductive traits in the Large White population. [file AGE-49-127-s002.pdf]

Table S1 Descriptive statistics of reproductive traits in the Large White population

Traits	N	Units	Mean	SD	Min	Max
TNB	1186	each	10.3	2.46	3	17
NBA	1170	each	9.91	2.36	3	17
LBW	1164	kg	13.36	3.28	3.6	24.6
ABW	1164	kg	1.36	0.18	0.89	2.09
GL	1175	day	115.11	2.17	100	127
AFS	1173	day	271.66	41.75	158	424
AFF	1155	day	386.96	41.95	273	540

N number of individuals in the association study, Mean arithmetic mean, SD standard deviation, Max maximum, Min minimum
