# Supplementary material for: Genome‐wide association study for reproductive traits in a Large White pig population
Source: Anim Genet. 2018 Feb 7;49(2):127–31. doi: 10.1111/age.12638 (PMC5873431; doi:10.1111/age.12638)
Supplement: Supplementary file 3 — Table S2 Suggestive significant SNPs for six reproductive traits. [file AGE-49-127-s003.pdf]

Table S2 Suggestive significant SNPs for six reproductive traits

Trait	SNP	Chr[a]	Position(bp)	*P* value	MAF[b]	β[c]	CPV%(SE)[d]	Nearest gene/Candidate Gene[e]	Location (bp)[f]
TNB	ASGA0099361	4	19239772	7.29E-06	0.34(G/A)	0.54	2.05(0.03)	*SNTB1*	177215
	WU_10.2_5_109775477	5	109775477	2.77E-05	0.12(A/G)	-0.66	1.45(0.02)	*ZDHHC17*	222364
	ASGA0093900	4	19237526	4.21E-05	0.24(G/A)	0.50	1.46(0.02)	*SNTB1*	179461
	WU_10.2_3_5435898	3	5435898	6.92E-05	0.43(G/A)	-0.43	1.43(0.02)	*LMTK2/**BHLHA15**/**OCM2***	within/**29060**/**129653**
	ASGA0003968	1	107465596	7.30E-05	0.15(A/G)	0.60	1.42(0.02)	*ZBTB7C/**SMAD2***	196317/**474096**
	ASGA0014296	3	44546555	7.64E-05	0.2(A/G)	-0.54	1.43(0.02)	*BARX1/**IL1B2***	57540/**623364**
	ALGA0073833	13	210866603	8.07E-05	0.37(A/G)	0.46	1.50(0.02)	*LOC106505861*	within
NBA	WU_10.2_3_44862084	3	44862084	6.06E-06	0.27(G/A)	-0.53	1.89(0.03)	*BARX1/**IL1B2***	254300/**307835**
	WU_10.2_14_135009946	14	135009946	3.13E-05	0.34(G/A)	-0.45	1.56(0.02)	*NRAP/**HABP2***	102219/**120035**
	ALGA0070192	13	58478836	6.39E-05	0.48(G/A)	0.41	1.44(0.02)	*EIF4E3*	292417
ABW	ASGA0020103	4	77339687	4.50E-06	0.16(A/G)	0.05	2.12(0.03)	*NKAIN3*	within
	ASGA0005237	1	185818261	1.61E-05	0.14(A/G)	0.05	2.03(0.03)	*RPLP1/**PAQR5***	431090/**615274**
	ALGA0121511	9	150097891	4.35E-05	0.31(A/G)	-0.04	1.72(0.03)	*DDC/**GRB10***	within/**170676**
	INRA0005103	1	184608591	4.58E-05	0.14(A/C)	0.05	1.85(0.03)	*ANP32A/**PAQR5***	within/**594396**
	DRGA0001605	1	186440796	4.87E-05	0.14(G/A)	0.05	1.82(0.03)	*UACA*	194928
	DRGA0012455	13	61231210	8.02E-05	0.44(A/C)	-0.04	2.02(0.03)	*CNTN3*	328217
GL	MARC0070353	15	132874095	1.49E-05	0.05(G/A)	-0.98	1.57(0.02)	*TNS1*	197013
	M1GA0007042	4	1231473	1.53E-05	0.16(A/G)	-0.63	1.59(0.02)	*ZC3H3/**GPIHBP1***	within/**113120**
	MARC0022818	15	132917763	3.87E-05	0.05(G/A)	-0.94	1.36(0.02)	*TNS1*	153345
	WU_10.2_2_149637913	2	149637913	4.13E-05	0.18(G/A)	-0.51	1.52(0.02)	*PCDH1*	41127
	WU_10.2_10_2982883	10	2982883	4.86E-05	0.05(G/A)	-0.89	1.55(0.02)	*RGS21*	27862
	H3GA0055313	2	154468364	6.60E-05	0.21(A/G)	-0.49	1.37(0.02)	*PPP2R2B/**PRELID2***	within/**849242**
	H3GA0014885	4	140338056	7.32E-05	0.09(A/C)	-0.66	1.24(0.02)	*PKN2*	403519
	WU_10.2_3_142997817	3	142997817	7.59E-05	0.26(A/G)	-0.44	1.48(0.02)	*LOC106508994*	within

Table S2 Cont

Trait	SNP	Chr[a]	Position(bp)	*P* value	MAF[b]	β[c]	CPV%(SE)[d]	Nearest gene/Candidate Gene[e]	Location (bp)[f]
GL	ASGA0059909	13	208055517	8.51E-05	0.33(G/A)	0.41	1.35(0.02)	*RCAN1*	21271
	M1GA0024771	6	39511968	8.73E-05	0.44(A/G)	-0.41	1.60(0.02)	*PDCD2L/**GPI/DMKN***	within/4852/**716399**
AFS	ASGA0097252	16	80412595	1.50E-05	0.38(G/A)	-0.20	1.89(0.03)	***ADCY2***	within
	DRGA0005679	5	38394071	3.56E-05	0.06(A/G)	0.39	1.79(0.03)	*TMEM19*	within
	DRGA0005681	5	38411675	3.56E-05	0.06(A/G)	0.39	1.79(0.03)	*TMEM19*	within
	WU_10.2_5_34798391	5	34798391	3.67E-05	0.18(A/G)	-0.24	1.70(0.03)	*GRIP1*	84162
	M1GA0014302	10	66852031	3.77E-05	0.09(A/G)	0.32	1.72(0.03)	*CELF2*	446674
	H3GA0053903	13	24423159	6.02E-05	0.43(A/C)	-0.19	1.68(0.02)	*LOC100514680/**ACAA1***	within/**745729**
	15_2154617	15	2154617	8.23E-05	0.36(G/A)	-0.18	1.42(0.02)	*LYPD6*	26625
AFF	M1GA0014302	10	66852031	2.06E-05	0.09(A/G)	0.33	1.84(0.03)	*CELF2*	446674
	ASGA0097252	16	80412595	2.26E-05	0.38(G/A)	-0.20	1.83(0.03)	***ADCY2***	**within**
	DRGA0005679	5	38394071	3.26E-05	0.07(A/G)	0.39	1.80(0.03)	*TMEM19*	within
	DRGA0005681	5	38411675	3.26E-05	0.07(A/G)	0.39	1.80(0.03)	*TMEM19*	within
	WU_10.2_5_34798391	5	34798391	7.55E-05	0.18(A/G)	-0.23	1.57(0.02)	*GRIP1*	84162
	ASGA0103106	3	16106430	8.20E-05	0.24(A/G)	-0.21	1.54(0.02)	*TYW1*	161723
	ALGA0109952	13	24941184	8.54E-05	0.43(G/A)	-0.18	1.58(0.02)	*CTDSPL /**ACVR2B***	within/**481044**
	WU_10.2_6_143137873	6	143137873	8.79E-05	0.39(A/C)	0.18	1.45(0.02)	*DAB1*	13879

[a] Pig Chromosome

[b] The allele frequency of first listed marker

[c] The allele substitution effect

[d] Contribution to phenotypic variance, standard error

[e] The gene name with bold type represents candidate genes with less than 1.0 Mb of the SNPs

[f] The location with bold type represents the distance between significant SNP and candidate gene
