# Supplementary material for: Genome‐wide association study for reproductive traits in a Large White pig population
Source: Anim Genet. 2018 Feb 7;49(2):127–31. doi: 10.1111/age.12638 (PMC5873431; doi:10.1111/age.12638)
Supplement: Supplementary file 4 — Table S3 Genotype–phenotype correlations of the most significant SNPs for four reproductive traits [file AGE-49-127-s004.pdf]

Table S3 Genotype–phenotype correlations of the most significant SNPs for four reproductive traits

Traits[a]	SNP[b]	P value	Genotypes/ Phenotype [c]	Genotype frequency	Mean ± SD[d]
TNB	WU_10.2_2_162527469	9.72E-07	AA(647)	0.55	10.58±2.47[A]
			AC(442)	0.37	10.14±2.38[B]
			CC(97)	0.08	9.2±2.37[AB]
NBA	WU_10.2_3_44631648	9.07E-07	AA(573)	0.49	10.08±2.33[A]
			AG(470)	0.4	9.92±2.38[B]
			GG(127)	0.11	9.07±2.29[B]
AFS	ALGA0111336	2.23E-06	AA(194)	0.17	266.42±36.12[B]
			AC(509)	0.43	269.37±40.73[B]
			CC(470)	0.4	276.3±44.42[A]
AFF	ALGA0111336	3.53E-06	AA(187)	0.16	381.6±36.46[B]
			AC(501)	0.43	384.63±40.91[B]
			CC(467)	0.41	391.61±44.51[A]

[a] t-test was implemented to show genotype-phenotype correlations with 4 traits

[b] means the most significant SNP for each trait

[c] means different genotypes and the number of phenotypes

[d] different letters (A,B,AB) in a column mean significant differences (P<0.01)
