# Supplementary material for: Genome‐wide association study for reproductive traits in a Large White pig population
Source: Anim Genet. 2018 Feb 7;49(2):127–31. doi: 10.1111/age.12638 (PMC5873431; doi:10.1111/age.12638)
Supplement: Supplementary file 5 — Table S4 Annotated genes with less than 1 Mb of significant SNPs. [file AGE-49-127-s005.pdf]

Table S4: Annotated genes with less than 1 Mb of significant SNPs

Traits	SNP	Chr	Position	*P*- value	Nearest gene	Location (bp)[a]	Adjacent genes (±1 Mb)
TNB	WU_10.2_2_162527469	2	162527469	9.72E-07	IFITM2	174050	ANO9 SIGIRR PKP3 SCGB1C1 ODF3 BET1L RIC8A SIRT3 PSMD13 NLRP6 IFITM5 IFITM1
	WU_10.2_3_44631648	3	44631648	1.19E-06	BARX1	23864	PHF2 FAM120A BICD2 TTL POLR1B CKAP2L NT5DC4 IL1B2 IL1B1 IL1A
	WU_10.2_3_44862084	3	44862084	2.83E-06	BARX1	254300	PHF2 FAM120A IL1B2 IL1B1 IL1A NT5DC4 CKAP2L POLR1B TTL ZC3H6 ZC3H8 FBLN7
	ALGA0098819	18	56535534	3.46E-06	LOC102165380	within	STK17A PSMA2 MRPL32 BLVRA POLM AEBP1 POLD2 MYL7 GCK YKT6 CAMK2B NUDCD3 DDX56 NPC1L1 TMED4 OGDH
	ASGA0099361	4	19239772	7.29E-06	SNTB1	177215	MTBP MRPL13 COL14A1 DEPTOR DSCC1 TAF2 SHAS2
	WU_10.2_5_109775477	5	109775477	2.77E-05	ZDHHC17	222364	CSRP2 E2F7 DYRK2 PPP6R2 SBF1 ADM2 MIOX LMF2 NCAPH2 ODF3B SCO2 KLHDC7B SYCE3 HDAC10 TUBGCP6 SELO TRABD PANX2 MOV10L1 MLC1
	ASGA0093900	4	19237526	4.21E-05	SNTB1	179461	MTBP MRPL13 COL14A1 DEPTOR DSCC1 TAF2 SHAS2
	WU_10.2_3_5435898	3	5435898	6.92E-05	LMTK2	within	BHLHA15 TECPR1 BRI3 BAIAP2L1 NPTX2 TMEM130 OCM2 CCZ1 RSPH10B PMS2 ANKRD61 TRRAP KPNA7 FSCN1 ACTB FBXL18 TNRC18 WIPI2
	ASGA0003968	1	107465596	7.30E-05	ZBTB7C	196317	SMAD2 CTIF SMAD7 RPL17
	ASGA0014296	3	44546555	7.64E-05	BARX1	57540	IL1B2 IL1B1 IL1A BICD2 FAM120A PHF2
	ALGA0073833	13	210866603	8.07E-05	LOC106505861	within	PIGP RIPPLY3 HLCS SIM2 CLDN14 CHAF1B MORC3 DOPEY2 DSCR3 DYRK1A
NBA	WU_10.2_3_44631648	3	44631648	9.07E-07	BARX1	23864	PHF2 FAM120A BICD2 TTL POLR1B CKAP2L NT5DC4 IL1B2 IL1B1 IL1A
	ALGA0098819	18	56535534	3.11E-06	LOC102165380	within	STK17A PSMA2 MRPL32 BLVRA POLM AEBP1 POLD2 MYL7 GCK YKT6 CAMK2B NUDCD3 DDX56 NPC1L1 TMED4 OGDH
	WU_10.2_2_162527469	2	162527469	4.42E-06	IFITM2	174050	ANO9 SIGIRR PKP3 SCGB1C1 ODF3 BET1L RIC8A SIRT3 PSMD13 NLRP6 IFITM5 IFITM1
	WU_10.2_3_44862084	3	44862084	6.06E-06	BARX1	254300	PHF2 FAM120A IL1B2 IL1B1 IL1A NT5DC4 CKAP2L POLR1B TTL ZC3H6 ZC3H8 FBLN7
	WU_10.2_14_135009946	14	135009946	3.14E-05	NRAP	102219	HABP2 DCLRE1A NHLRC2 ADRB1 CCDC186 TDRD1 VWA2 AFAP1L2 ABLIM1 TCF7L2
	ALGA0070192	13	58478836	6.40E-05	EIF4E3	292417	PROK2 RYBP FOXP1

Traits	SNP	Chr	Position	*P*- value	Nearest gene	Location (bp)[a]	Adjacent genes (±1 Mb)
**ABW**	ASGA0020103	4	77339687	4.53E-06	NKAIN3	within	GGH TTPA YTHDF3
	ASGA0005237	1	185818261	1.61E-05	RPLP1	431090	KIF23 PAQR5 GLCE NOX5 UACA LARP6 LRRC49
	ALGA0121511	9	150097891	4.35E-05	DDC	within	GRB10 COBL FIGNL1 C9H7orf72 ZPBP VWC2
	INRA0005103	1	184608591	4.58E-05	ANP32A	within	NOX5 PAQR5 KIF23 RPLP1 ITGA11 FEM1B CLN6 PIAS1
	DRGA0001605	1	186440796	4.87E-05	UACA	194928	LARP6 THAP10 LRRC49
	DRGA0012455	13	61231210	8.02E-05	CNTN3	328217	PDZRN3
**AFS**	ALGA0111336	16	80445920	2.23E-06	ADCY2	within	FASTKD3 MTRR
	ASGA0097252	16	80412595	1.50E-05	ADCY2	within	FASTKD3 MTRR
	DRGA0005679	5	38394071	3.56E-05	TMEM19	within	THAP2 LGR5 TSPAN8 PTPRR RAB21 TBC1D15 TPH2 TRHDE
	DRGA0005681	5	38411675	3.56E-05	TMEM19	within	THAP2 LGR5 TSPAN8 PTPRR RAB21 TBC1D15 TPH2 TRHDE
	WU_10.2_5_34798391	5	34798391	3.67E-05	GRIP1	84162	CAND1 IL26 IFNG MDM1 RAP1B NUP107 HELB IRAK3 TMBIM4
	M1GA0014302	10	66852031	3.77E-05	CELF2	446674	USP6NL ECHDC3
	H3GA0053903	13	24423159	6.02E-05	LOC100514680	within	GOLGA4 EPM2AIP1 DCLK3 LRRFIP2 MLH1 STAC PLCD1 VILL CTDSPL DLEC1 ACAA1 MYD88 OXSR1 XYLB SLC22A14
	15_2154617	15	2154617	8.23E-05	LYPD6	26625	MMADHC LYPD6B KIF5C EPC2
**AFF**	ALGA0111336	16	80445920	3.53E-06	ADCY2	within	FASTKD3 MTRR
	M1GA0014302	10	66852031	2.06E-05	CELF2	446674	USP6NL ECHDC3
	ASGA0097252	16	80412595	2.26E-05	ADCY2	within	FASTKD3 MTRR
	DRGA0005679	5	38394071	3.26E-05	TMEM19	within	THAP2 LGR5 TSPAN8 PTPRR RAB21 TBC1D15 TPH2 TRHDE
	DRGA0005681	5	38411675	3.26E-05	TMEM19	within	THAP2 LGR5 TSPAN8 PTPRR RAB21 TBC1D15 TPH2 TRHDE
	WU_10.2_5_34798391	5	34798391	7.55E-05	GRIP1	84162	CAND1 IL26 IFNG MDM1 RAP1B NUP107 HELB IRAK3 TMBIM4
	ASGA0103106	3	16106430	8.20E-05	TYW1	161723	SBDS TMEM248 RABGEF1 KCTD7 TPST1 CRCP ASL GUSB VKORC1L1 NUPR1L CHCHD2 PHKG1 SUMF2 CCT6A CALN1 WBSCR17
	ALGA0109952	13	24941184	8.54E-05	CTDSPL	within	DLEC1 ACAA1 MYD88 OXSR1 XYLB SLC22A14 ACVR2B EXOG SCN5A DCLK3 LRRFIP2 MLH1 STAC PLCD1 VILL
	WU_10.2_6_143137873	6	143137873	8.79E-05	DAB1	13879	C8B C8A PRKAA2 PPAP2B

Traits	SNP	Chr	Position	*P*- value	Nearest gene	Location (bp)[a]	Adjacent genes (±1 Mb)
GL	ALGA0061535	11	25305148	1.42E-06	AKAP11	99469	DGKH VWA8 RGCC NAA16 TNFSF11 FAM216B EPSTI1 DNAJC15
	WU_10.2_4_1247716	4	1247716	3.32E-06	ZC3H3	14516	GLI4 GPIHBP1 LY6H TOP1MT ZNF696 MROH5 GPR20 SLC45A4 PTK2 MAFA RHPN1 GSDMD MROH6 EEF1D PYCRL CCDC166 EXOSC4 PARP10 GPAA1 SHARPIN MAF1 HGH1 BOP1 MROH1 HSF1 DGAT1 SCRT1 SPATC1 SLC52A2 FBXL6 TMEM249 ADCK5 CPSF1 SLC39A4 VPS28 TONSL CYHR1 KIFC2 FOXH1 PPP1R16A GPT MFSD3 RECQL4 LRRC14 ARHGAP39
	ASGA0023643	4	1166037	3.87E-06	MAFA	7303	ZNF34 RPL8 RPL8 COMMD5
	MARC0070353	15	132874095	1.49E-05	TNS1	197013	RUFY4 CXCR2 CXCR1 PNKD ARPC2 TMBIM1 AAMP GPBAR1 CATIP SLC11A1 VIL1 USP37 RQCD1 PLCD4 PRKAG3 CYP27A1 RNF25
	M1GA0007042	4	1231473	1.53E-05	ZC3H3	within	GLI4 GPIHBP1 LY6H TOP1MT ZNF696 MROH5 GPR20 SLC45A4 PTK2 MAFA RHPN1 GSDMD MROH6 EEF1D PYCRL CCDC166 EXOSC4 PARP10 GPAA1 SHARPIN MAF1 HGH1 BOP1 MROH1 HSF1 DGAT1 SCRT1 SPATC1 SLC52A2 FBXL6 TMEM249 ADCK5 CPSF1 SLC39A4 VPS28 TONSL CYHR1 KIFC2 FOXH1 PPP1R16A GPT MFSD3 RECQL4 LRRC14 ARHGAP39
	MARC0022818	15	132917763	3.87E-05	TNS1	153345	GLI4 GPIHBP1 LY6H TOP1MT ZNF696 MROH5 GPR20 SLC45A4 PTK2 MAFA RHPN1 GSDMD MROH6 EEF1D PYCRL CCDC166 EXOSC4 PARP10 GPAA1 SHARPIN MAF1 HGH1 BOP1 MROH1 HSF1 DGAT1 SCRT1 SPATC1 SLC52A2 FBXL6 TMEM249 ADCK5 CPSF1 SLC39A4 VPS28 TONSL CYHR1 KIFC2 FOXH1 PPP1R16A GPT MFSD3 RECQL4 LRRC14 ARHGAP39
	WU_10.2_2_149637913	2	149637913	4.13E-05	PCDH1	41127	KIAA0141 PCDH12 RNF14 GNPDA1 NDFIP1 SPRY4 FGF1 ARHGAP26 ARAP3 FCHSD1 RELL2 HDAC3 DIAPH1 TAF7 PCDHB1 PCDHA11
	WU_10.2_10_2982883	10	2982883	4.86E-05	RGS21	27862	RGS2 UCHL5 B3GALT2 RGS18
	H3GA0055313	2	154468364	6.60E-05	PPP2R2B	within	JAKMIP2 GPR151 TCERG1 POU4F3 RBM27 PLAC8L1 SH3RF2 LARS GRXCR2 PRELID2
	H3GA0014885	4	140338056	7.32E-05	PKN2	403519	GTF2B CCBL2 GBP1 GBP2 LMO4
	WU_10.2_3_142997817	3	142997817	7.59E-05	LOC106508994	within	FAM84A
	ASGA0059909	13	208055517	8.51E-05	RCAN1	21271	CLIC6 RUNX1 KCNE1 C13H21orf140 SMIM11A KCNE2 MRPS6 SLC5A3 ATP5O CRYZL1 DONSON
	M1GA0024771	6	39511968	8.73E-05	PDCD2L	within	GPI UBA2 ZNF181 ZNF792 GRAMD1A SCN1B HPN LGI4 FXYD3 FXYD5 FAM187B LSR USF2 HAMP MAG DMKN KRTDAP FFAR2 FFAR3 FFAR1 SBSN GAPDHS ATP4A KIAA0355 LSM14A CEBPG CEBPA SLC7A10 LRP3 WDR88
