# Supplementary material for: Genome‐wide association study for reproductive traits in a Large White pig population
Source: Anim Genet. 2018 Feb 7;49(2):127–31. doi: 10.1111/age.12638 (PMC5873431; doi:10.1111/age.12638)
Supplement: Supplementary file 6 — Table S5 Significant Gene Ontology terms for five reproductive traits. [file AGE-49-127-s006.pdf]

Table S5: Significant Gene Ontology terms for five reproductive traits (*P*-value < 0.05)						
Traits	Category	GO term	GO term description	Counts	Invovled genes[a]	DAVID *P*-value
**TNB**	Biological Process	GO:0009880	embryonic pattern specification	3	SIM2, **SMAD2,** RIPPLY3	0.005982671
	Biological Process	GO:0001660	fever generation	2	IL1A, **IL1B2**	0.015114436
	Biological Process	GO:0008285	negative regulation of cell proliferation	7	IL1A, MTBP, E2F7, **SMAD2**, RIPPLY3, **IL1B2,** IFITM2	0.017553851
	Biological Process	GO:0006468	protein phosphorylation	7	DYRK2, ADM2, CAMK2B, MORC3, **SMAD2**, DYRK1A, STK17A	0.028366901
	Biological Process	GO:0071639	positive regulation of monocyte chemotactic protein-1 production	2	IL1A, **IL1B2**	0.034916353
	Biological Process	GO:0030213	hyaluronan biosynthetic process	2	SHAS2, **IL1B2**	0.034916353
	Biological Process	GO:0035234	ectopic germ cell programmed cell death	2	IL1A, **IL1B2**	0.039805036
**NBA**	Biological Process	GO:0032024	positive regulation of insulin secretion	3	TCF7L2, **GCK**, SIRT3	0.005898914
	Biological Process	GO:0001660	fever generation	2	IL1A, **IL1B2**	0.008198628
	Biological Process	GO:0071639	positive regulation of monocyte chemotactic protein-1 production	2	IL1A, **IL1B2**	0.019027916
	Biological Process	GO:0006954	inflammatory response	5	IL1A, AFAP1L2, PROK2, NLRP6, **IL1B2**	0.020492973
	Biological Process	GO:0035234	ectopic germ cell programmed cell death	2	IL1A, **IL1B2**	0.021717112
	Biological Process	GO:0045086	positive regulation of interleukin-2 biosynthetic process	2	IL1A, **IL1B2**	0.032401965
	Biological Process	GO:0000165	MAPK cascade	4	PSMD13, CAMK2B, PSMA2, **IL1B2**	0.034535979
	Biological Process	GO:0009607	response to biotic stimulus	2	IFITM5, IFITM2	0.037701495
**AFS**	Biological Process	GO:0032747	positive regulation of interleukin-23 production	2	MYD88, IFNG	0.011268493
	Biological Process	GO:0035556	intracellular signal transduction	5	OXSR1, GRIP1, STAC, **ADCY2**, PLCD1	0.013224442
	Biological Process	GO:0045080	positive regulation of chemokine biosynthetic process	2	MYD88, IFNG	0.022413312
	Biological Process	GO:0044130	negative regulation of growth of symbiont in host	2	MYD88, IFNG	0.033435778

Traits	Category	GO term	GO term description	Counts	Invovled genes[a]	DAVID P-value
**AFF**	Biological Process	GO:0035556	intracellular signal transduction	6	OXSR1, PRKAA2, GRIP1, STAC, **ADCY2,** PLCD1	0.009123888
	Biological Process	GO:0032747	positive regulation of interleukin-23 production	2	MYD88, IFNG	0.015688517
	Biological Process	GO:0045080	positive regulation of chemokine biosynthetic process	2	MYD88, IFNG	0.031135477
	Biological Process	GO:0006957	complement activation, alternative pathway	2	C8A, C8B	0.040289226
	Biological Process	GO:0044130	negative regulation of growth of symbiont in host	2	MYD88, IFNG	0.046344529
**GL**	Biological Process	GO:0038112	interleukin-8-mediated signaling pathway	2	CXCR2, CXCR1	0.014955723
	Biological Process	GO:0030041	actin filament polymerization	3	DIAPH1, CATIP, VIL1	0.0149789
	Biological Process	GO:0006366	transcription from RNA polymerase II promoter	10	POU4F3, LMO4, TAF7, FOXH1, GTF2B, USF2, MAFA, TCERG1, CEBPG, CEBPA	0.015973518
	Biological Process	GO:0008360	regulation of cell shape	5	DIAPH1, ARAP3, PTK2, HPN, VIL1	0.020734949
	Biological Process	GO:0045944	positive regulation of transcription from RNA polymerase II promoter	14	FGF1, POU4F3, FOXH1, SLC11A1, HDAC3, USF2, MAFA, CEBPG, LMO4, TAF7, RGCC, HSF1, TNFSF11, CEBPA	0.034377019
	Biological Process	GO:0042593	glucose homeostasis	4	FFAR2, **GPI**, FFAR1, CEBPA	0.043504194
	Biological Process	GO:0009299	mRNA transcription	2	HSF1, EEF1D	0.044204661
	Biological Process	GO:1902533	positive regulation of intracellular signal transduction	2	FGF, TNFSF11	0.044204661
	Biological Process	GO:0006879	cellular iron ion homeostasis	3	SLC11A1, HAMP, NDFIP1	0.046681401

[a]: gene name with bold type represents candidate genes that involved in this biological Process
