# Supplementary material for: Genome‐wide association study for reproductive traits in a Large White pig population
Source: Anim Genet. 2018 Feb 7;49(2):127–31. doi: 10.1111/age.12638 (PMC5873431; doi:10.1111/age.12638)
Supplement: Supplementary file 7 — Table S6 Significant SNPs located in known QTL regions for reproductive traits. [file AGE-49-127-s007.pdf]

Table S6: Significant SNPs located in known QTL regions for reproductive traits

SNP[a]	Position	Chromosome	Database	QTL style	Start	End	QTL_ID	Trait	PUBMED_ID
ASGA0003968	107465596	Chr.1	Animal QTLdb	Reproduction_QTL	85842604	162073157	6481	Teat number	19226448
ASGA0003968	107465596	Chr.1	Animal QTLdb	Reproduction_QTL	105873529	290457491	5255	Teat number	18651874
ASGA0003968	107465596	Chr.1	Animal QTLdb	Reproduction_QTL	45939845	292033322	5223	Teat number	18651874
ASGA0005237	185818261	Chr.1	Animal QTLdb	Reproduction_QTL	181896016	186896016	106223	Litter size	27317562
ASGA0005237	185818261	Chr.1	Animal QTLdb	Reproduction_QTL	105873529	290457491	5255	Teat number	18651874
ASGA0005237	185818261	Chr.1	Animal QTLdb	Reproduction_QTL	45939845	292033322	5223	Teat number	18651874
ASGA0005237	185818261	Chr.1	Animal QTLdb	Reproduction_QTL	165580236	257944209	7453	Nonfunctional nipples	18219525
INRA0005103	184608591	Chr.1	Animal QTLdb	Reproduction_QTL	181896016	186896016	106223	Litter size	27317562
INRA0005103	184608591	Chr.1	Animal QTLdb	Reproduction_QTL	105873529	290457491	5255	Teat number	18651874
INRA0005103	184608591	Chr.1	Animal QTLdb	Reproduction_QTL	45939845	292033322	5223	Teat number	18651874
INRA0005103	184608591	Chr.1	Animal QTLdb	Reproduction_QTL	165580236	257944209	7453	Nonfunctional nipples	18219525
DRGA0001605	186440796	Chr.1	Animal QTLdb	Reproduction_QTL	181896016	186896016	106223	Litter size	27317562
DRGA0001605	186440796	Chr.1	Animal QTLdb	Reproduction_QTL	105873529	290457491	5255	Teat number	18651874
DRGA0001605	186440796	Chr.1	Animal QTLdb	Reproduction_QTL	45939845	292033322	5223	Teat number	18651874
DRGA0001605	186440796	Chr.1	Animal QTLdb	Reproduction_QTL	165580236	257944209	7453	Nonfunctional nipples	18219525
WU_10.2_2_149637913	149637913	Chr.2	Animal QTLdb	Reproduction_QTL	139359663	150135089	4255	Nonfunctional nipples	17032781
WU_10.2_3_44631648	44631648	Chr.3	Animal QTLdb	Reproduction_QTL	4571903	82495610	515	Corpus luteum number	10375216
WU_10.2_3_44631648	44631648	Chr.3	Animal QTLdb	Reproduction_QTL	30144110	87077830	6465	Left teat number	19226448
WU_10.2_3_44631648	44631648	Chr.3	Animal QTLdb	Reproduction_QTL	44546555	45819174	37438	Teat number	24981054
WU_10.2_3_44631648	44631648	Chr.3	Animal QTLdb	Reproduction_QTL	14776389	102773204	7455	Nonfunctional nipples	18219525
WU_10.2_3_44631648	44631648	Chr.3	Animal QTLdb	Reproduction_QTL	14776389	100216075	7472	Nonfunctional nipples	18219525
WU_10.2_3_44631648	44631648	Chr.3	Animal QTLdb	Reproduction_QTL	1456046	130209174	5224	Teat number	18651874
WU_10.2_3_44862084	44862084	Chr.3	Animal QTLdb	Reproduction_QTL	4571903	82495610	515	Corpus luteum number	10375216
WU_10.2_3_44862084	44862084	Chr.3	Animal QTLdb	Reproduction_QTL	30144110	87077830	6465	Left teat number	19226448
WU_10.2_3_44862084	44862084	Chr.3	Animal QTLdb	Reproduction_QTL	44546555	45819174	37438	Teat number	24981054
WU_10.2_3_44862084	44862084	Chr.3	Animal QTLdb	Reproduction_QTL	14776389	102773204	7455	Nonfunctional nipples	18219525
WU_10.2_3_44862084	44862084	Chr.3	Animal QTLdb	Reproduction_QTL	14776389	100216075	7472	Nonfunctional nipples	18219525
WU_10.2_3_44862084	44862084	Chr.3	Animal QTLdb	Reproduction_QTL	1456046	130209174	5224	Teat number	18651874

SNP[a]	Position	Chromosome	Database	QTL style	Start	End	QTL_ID	Trait	PUBMED_ID
WU_10.2_3_5435898	5435898	Chr.3	Animal QTLdb	Reproduction_QTL	4571903	82495610	515	Corpus luteum number	10375216
WU_10.2_3_5435898	5435898	Chr.3	Animal QTLdb	Reproduction_QTL	1456046	130209174	5224	Teat number	18651874
ASGA0014296	44546555	Chr.3	Animal QTLdb	Reproduction_QTL	4571903	82495610	515	Corpus luteum number	10375216
ASGA0014296	44546555	Chr.3	Animal QTLdb	Reproduction_QTL	30144110	87077830	6465	Left teat number	19226448
ASGA0014296	44546555	Chr.3	Animal QTLdb	Reproduction_QTL	14776389	102773204	7455	Nonfunctional nipples	18219525
ASGA0014296	44546555	Chr.3	Animal QTLdb	Reproduction_QTL	14776389	100216075	7472	Nonfunctional nipples	18219525
ASGA0014296	44546555	Chr.3	Animal QTLdb	Reproduction_QTL	1456046	130209174	5224	Teat number	18651874
WU_10.2_3_142997817	142997817	Chr.3	Animal QTLdb	Reproduction_QTL	125343844	143758669	4250	Teat number	17032781
WU_10.2_3_142997817	142997817	Chr.3	Animal QTLdb	Reproduction_QTL	125343844	143758669	4256	Nonfunctional nipples	17032781
ASGA0103106	16106430	Chr.3	Animal QTLdb	Reproduction_QTL	14776389	16866926	4249	Corpus luteum number	17032781
ASGA0103106	16106430	Chr.3	Animal QTLdb	Reproduction_QTL	4571903	82495610	515	Corpus luteum number	10375216
ASGA0103106	16106430	Chr.3	Animal QTLdb	Reproduction_QTL	14776389	102773204	7455	Nonfunctional nipples	18219525
ASGA0103106	16106430	Chr.3	Animal QTLdb	Reproduction_QTL	14776389	100216075	7472	Nonfunctional nipples	18219525
ASGA0103106	16106430	Chr.3	Animal QTLdb	Reproduction_QTL	1456046	130209174	5224	Teat number	18651874
ASGA0099361	19239772	Chr.4	Animal QTLdb	Reproduction_QTL	5244479	95136118	5256	Teat number	18651874
ASGA0099361	19239772	Chr.4	Animal QTLdb	Reproduction_QTL	4064787	71152561	5225	Teat number	18651874
ASGA0093900	19237526	Chr.4	Animal QTLdb	Reproduction_QTL	5244479	95136118	5256	Teat number	18651874
ASGA0093900	19237526	Chr.4	Animal QTLdb	Reproduction_QTL	4064787	71152561	5225	Teat number	18651874
ASGA0020103	77339687	Chr.4	Animal QTLdb	Reproduction_QTL	75991872	79645252	55865	Cryptorchidism	25886970
ASGA0020103	77339687	Chr.4	Animal QTLdb	Reproduction_QTL	5244479	95136118	5256	Teat number	18651874
ASGA0020103	77339687	Chr.4	Animal QTLdb	Reproduction_QTL	23809394	82084284	8817	Corpus luteum number	22443659
H3GA0014885	140338056	Chr.4	Animal QTLdb	Reproduction_QTL	102054630	140378854	7473	Nonfunctional nipples	18219525
DRGA0005679	38394071	Chr.5	Animal QTLdb	Reproduction_QTL	231749	108789684	18128	Number of stillborn	22221021
DRGA0005679	38394071	Chr.5	Animal QTLdb	Reproduction_QTL	11496289	77598894	24288	Teat number	24456574
DRGA0005679	38394071	Chr.5	Animal QTLdb	Reproduction_QTL	22412792	63117217	10619	uterine horn length	10341088
DRGA0005679	38394071	Chr.5	Animal QTLdb	Reproduction_QTL	33251630	69110631	18378	uterine horn weight	21948608
DRGA0005679	38394071	Chr.5	Animal QTLdb	Reproduction_QTL	33251630	69110631	18380	Reproductive tract weight	21948608
DRGA0005681	38411675	Chr.5	Animal QTLdb	Reproduction_QTL	231749	108789684	18128	Number of stillborn	22221021
DRGA0005681	38411675	Chr.5	Animal QTLdb	Reproduction_QTL	11496289	77598894	24288	Teat number	24456574
DRGA0005681	38411675	Chr.5	Animal QTLdb	Reproduction_QTL	22412792	63117217	10619	uterine horn length	10341088
DRGA0005681	38411675	Chr.5	Animal QTLdb	Reproduction_QTL	33251630	69110631	18378	uterine horn weight	21948608
DRGA0005681	38411675	Chr.5	Animal QTLdb	Reproduction_QTL	33251630	69110631	18380	Reproductive tract weight	21948608

SNP[a]	Position	Chromosome	Database	QTL style	Start	End	QTL_ID	Trait	PUBMED_ID
WU_10.2_5_34798391	34798391	Chr.5	Animal QTLdb	Reproduction_QTL	231749	108789684	18128	Number of stillborn	22221021
WU_10.2_5_34798391	34798391	Chr.5	Animal QTLdb	Reproduction_QTL	11496289	77598894	24288	Teat number	24456574
WU_10.2_5_34798391	34798391	Chr.5	Animal QTLdb	Reproduction_QTL	22412792	63117217	10619	uterine horn length	10341088
WU_10.2_5_34798391	34798391	Chr.5	Animal QTLdb	Reproduction_QTL	33251630	69110631	18378	uterine horn weight	21948608
WU_10.2_5_34798391	34798391	Chr.5	Animal QTLdb	Reproduction_QTL	33251630	69110631	18380	Reproductive tract weight	21948608
M1GA0024771	39511968	Chr.6	Animal QTLdb	Reproduction_QTL	2352681	72944202	24289	Teat number	24456574
M1GA0024771	39511968	Chr.6	Animal QTLdb	Reproduction_QTL	19536155	157765593	5226	Teat number	18651874
M1GA0024771	39511968	Chr.6	Animal QTLdb	Reproduction_QTL	38553933	66465212	7459	Nonfunctional nipples	18219525
M1GA0024771	39511968	Chr.6	Animal QTLdb	Reproduction_QTL	9303695	77907573	24281	Litter size	24456574
WU_10.2_6_143137873	143137873	Chr.6	Animal QTLdb	Reproduction_QTL	19536155	157765593	5226	Teat number	18651874
WU_10.2_6_143137873	143137873	Chr.6	Animal QTLdb	Reproduction_QTL	91030131	146365886	8820	Age at puberty	22443659
M1GA0014302	66852031	Chr.10	Animal QTLdb	Reproduction_QTL	25917876	67676064	518	Corpus luteum number	10375216
M1GA0014302	66852031	Chr.10	Animal QTLdb	Reproduction_QTL	61209673	72237308	1107	Teat number	ISU0016
M1GA0014302	66852031	Chr.10	Animal QTLdb	Reproduction_QTL	66332574	73332866	521	Age at puberty	10375216
ALGA0061535	25305148	Chr.11	Animal QTLdb	Reproduction_QTL	1111096	68683528	5260	Teat number	18651874
ALGA0070192	58478836	Chr.13	Animal QTLdb	Reproduction_QTL	17040484	120146164	18134	Number of stillborn	22221021
ALGA0070192	58478836	Chr.13	Animal QTLdb	Reproduction_QTL	5836383	188271972	7479	Nonfunctional nipples	18219525
ALGA0070192	58478836	Chr.13	Animal QTLdb	Reproduction_QTL	18268056	206704152	24285	Corpus luteum number	24456574
ALGA0070192	58478836	Chr.13	Animal QTLdb	Reproduction_QTL	27210688	91206297	7466	Nonfunctional nipples	18219525
DRGA0012455	61231210	Chr.13	Animal QTLdb	Reproduction_QTL	17040484	120146164	18134	Number of stillborn	22221021
DRGA0012455	61231210	Chr.13	Animal QTLdb	Reproduction_QTL	5836383	188271972	7479	Nonfunctional nipples	18219525
DRGA0012455	61231210	Chr.13	Animal QTLdb	Reproduction_QTL	18268056	206704152	24285	Corpus luteum number	24456574
DRGA0012455	61231210	Chr.13	Animal QTLdb	Reproduction_QTL	27210688	91206297	7466	Nonfunctional nipples	18219525
H3GA0053903	24423159	Chr.13	Animal QTLdb	Reproduction_QTL	17040484	120146164	18134	Number of stillborn	22221021
H3GA0053903	24423159	Chr.13	Animal QTLdb	Reproduction_QTL	88939	27210688	493	Corpus luteum number	9250508
H3GA0053903	24423159	Chr.13	Animal QTLdb	Reproduction_QTL	5836383	188271972	7479	Nonfunctional nipples	18219525
H3GA0053903	24423159	Chr.13	Animal QTLdb	Reproduction_QTL	9843648	45496370	8824	Age at puberty	22443659
H3GA0053903	24423159	Chr.13	Animal QTLdb	Reproduction_QTL	9843648	45496370	8826	Corpus luteum number	22443659
H3GA0053903	24423159	Chr.13	Animal QTLdb	Reproduction_QTL	18268056	206704152	24285	Corpus luteum number	24456574

SNP[a]	Position	Chromosome	Database	QTL style	Start	End	QTL_ID	Trait	PUBMED_ID
ALGA0109952	24941184	Chr.13	Animal QTLdb	Reproduction_QTL	17040484	120146164	18134	Number of stillborn	22221021
ALGA0109952	24941184	Chr.13	Animal QTLdb	Reproduction_QTL	88939	27210688	493	Corpus luteum number	9250508
ALGA0109952	24941184	Chr.13	Animal QTLdb	Reproduction_QTL	5836383	188271972	7479	Nonfunctional nipples	18219525
ALGA0109952	24941184	Chr.13	Animal QTLdb	Reproduction_QTL	9843648	45496370	8824	Age at puberty	22443659
ALGA0109952	24941184	Chr.13	Animal QTLdb	Reproduction_QTL	9843648	45496370	8826	Corpus luteum number	22443659
ALGA0109952	24941184	Chr.13	Animal QTLdb	Reproduction_QTL	18268056	206704152	24285	Corpus luteum number	24456574
MARC0070353	132874095	Chr.15	Animal QTLdb	Reproduction_QTL	53604367	140194479	519	Corpus luteum number	10375216
MARC0070353	132874095	Chr.15	Animal QTLdb	Reproduction_QTL	33137272	149797711	7468	Nonfunctional nipples	18219525
MARC0070353	132874095	Chr.15	Animal QTLdb	Reproduction_QTL	127880313	135791936	494	Corpus luteum number	9250508
MARC0022818	132917763	Chr.15	Animal QTLdb	Reproduction_QTL	53604367	140194479	519	Corpus luteum number	10375216
MARC0022818	132917763	Chr.15	Animal QTLdb	Reproduction_QTL	33137272	149797711	7468	Nonfunctional nipples	18219525
MARC0022818	132917763	Chr.15	Animal QTLdb	Reproduction_QTL	127880313	135791936	494	Corpus luteum number	9250508
ALGA0098819	56535534	Chr.18	Animal QTLdb	Reproduction_QTL	52307963	77675048	18236	Litter size	22221021
ALGA0098819	56535534	Chr.18	Animal QTLdb	Reproduction_QTL	32798519	61220071	7470	Nonfunctional nipples	18219525

[a]: 29 different significant SNPs in this study
